# Effects of Hot Balloon vs. Cryoballoon Ablation for Atrial Fibrillation: A Systematic Review, Meta-Analysis, and Meta-Regression

**DOI:** 10.3389/fcvm.2021.787270

**Published:** 2021-12-15

**Authors:** Xinyi Peng, Xiao Liu, Hongbo Tian, Yu Chen, Xuexun Li

**Affiliations:** ^1^Heart Center, Beijing Chao-Yang Hospital, Capital Medical University, Beijing, China; ^2^Department of Cardiology, Qingdao University Medical College Affiliated Yantai Yuhuangding Hospital, Yantai, China; ^3^Department of Cardiology, Shandong Provincial Hospital Affiliated to Shandong First Medical University, Jinan, China; ^4^Peking University International Hospital, Beijing, China

**Keywords:** catheter ablation, cryoballoon ablation, hot balloon ablation, atrial fibrillation, prognosis, meta-analysis

## Abstract

**Background:** Balloon-based catheter ablations, including hot balloon ablation (HBA) and cryoballoon ablation (CBA), have rapidly emerged as alternative modalities to conventional catheter atrial fibrillation (AF) ablation owing to their impressive procedural advantages and better clinical outcomes and safety. However, the differences in characteristics, effectiveness, safety, and efficacy between HBA and CBA remain undetermined. This study compares the characteristic and prognosis differences between HBA and CBA.

**Methods:** Electronic search was conducted in six databases (PubMed, Embase, Cochrane Library, Web of Science, ClinicalTrial.gov, and medRxiv) with specific search strategies. Eligible studies were selected based on specific criteria; all records were identified up to June 1, 2021. The mean difference, odds ratios (ORs), and 95% confidence intervals (CIs) were calculated to evaluate the clinical outcomes. Heterogeneity and risk of bias were assessed using predefined criteria.

**Results:** Seven studies were included in the final meta-analysis. Compared with CBA, more patients in the HBA group had residual conduction and required a higher incidence of touch-up ablation (TUA) [OR (95% CI) = 2.76 (2.02–3.77), *P* = 0.000]. The most frequent sites of TUA were the left superior pulmonary veins (PVs) in the HBA group vs. the right inferior PVs in the CBA group. During HBA surgery, the left and right superior PVs were more likely to have a higher fluid injection volume. Furthermore, the procedure time was longer in the HBA group than in the CBA group [weighted mean difference (95% CI) = 14.24 (4.39–24.09), *P* = 0.005]. Patients in the CBA group could have an increased risk of AF occurrence, and accepted more antiarrhythmic drug therapy; however, the result was insignificant.

**Conclusions:** HBA and CBA are practical ablation approaches for AF treatment. Patients who received HBA had a higher incidence of TUA and longer procedure time. Clinical outcomes during the mid-term follow-up between HBA and CBA were comparable.

**Systematic Review Registration:** https://www.crd.york.ac.uk/PROSPERO/display_record.php?RecordID=259487, identifier: CRD42021259487.

## Introduction

Catheter ablation has been the most effective therapeutic approach and has been awarded the highest-level guideline recommendation for the treatment of atrial fibrillation (AF) since the decades (1). Pulmonary vein (PV) isolation is the most commonly used, and fundamental strategy in contemporary clinical practice. Several balloon-based catheter ablations, including hot balloon ablation (HBA) and cryoballoon ablation (CBA), have rapidly emerged as alternatives to conventional radiofrequency (RF) catheter-based AF ablation. As previous studies have reported, balloon-based ablations not only have equal clinical utility (2, 3) but also provide several advantages over traditional surgery, such as a more extensive wide-area ablation (4, 5), shorter procedure duration (6), and less frequent dormant PV conduction (7). Moreover, compared with antiarrhythmic drug therapy, balloon-based ablations possess the advantage of reducing AF recurrence and improving the patients' quality of life without increasing the incidence of adverse events (8, 9). However, the differences in characteristics, effectiveness, safety, and efficacy between HBA and CBA remain undetermined. This meta-analysis aimed to compare these two approaches in AF management and guide the optimum selection of balloon-based catheter ablation as the initial rhythm control strategy in patients with AF during routine clinical practice.

## Methods

### Search Strategy

This work was registered in the International Prospective Register of Systematic Reviews, and was identified as CRD42021259487. Our electronic search was conducted in six databases, including PubMed, Embase, Cochrane Library, Web of Science, ClinicalTrial.gov, and medRxiv, with specific search strategies using keywords “[Cryosurgery OR Cryoablation OR cryoballoon] AND [HotBalloon OR thermal balloon OR radiofrequency thermal balloon catheter OR hot balloon] AND [Atrial Fibrillation].” Results from the time of database establishment to June 1, 2021 were included. We also analyzed reference lists of relevant studies to identify potentially eligible articles. No restrictions on language were applied. The search strategies are listed in Supplementary Table 1.

### Study Selection

Eligible studies were selected according to the following inclusion criteria: (1) prospective or retrospective cohort studies, (2) studies of adult patients diagnosed with AF treated with HBA or CBA; and (3) studies reporting surgical complications and clinical outcomes. Exclusion criteria were (1) studies with inaccessible or incomplete full texts, and (2) those with incomplete data reports. Our analysis did not include duplicate articles, conference abstracts, case reports, review articles, comments, letters, animal studies, or *in vitro* studies. Using the above criteria, two authors (Peng and Chen) independently reviewed all the articles by browsing abstracts and titles for selecting relevant studies, which were then subjected to further screening. In case of any difference in opinion or disagreement between the two authors, the corresponding author (Li) was consulted.

### Literature Quality Evaluation

The quality and bias of cohort studies were assessed using the original Newcastle-Ottawa Scale (NOS). The total score was 9; studies with 4 points or less were considered low-quality literature, while those with 5 points or more were deemed high-quality.

### Data Extraction and Statistical Analyses

Data from all eligible studies were extracted by one author (Peng) and given to another author (Chen) for cross-checking. Extracted data included the following: first author's name, date of publication, type of study, study location, study characteristics (sample size, sex, age, follow-up days, comorbidities, baseline characteristics), clinical manifestations, surgical characteristics, complications, and clinical outcomes. Data analysis was conducted using Stata software version 15.0, and pooled using the fixed-effects models; if the heterogeneity was significant, random-effect models were applied. Results of data analysis were odds ratios (ORs) and 95% confidence intervals (CIs) for dichotomous variables, weighted mean difference (WMD), and 95% CI for continuous outcomes. Finally, tests of heterogeneity were performed using the *I*^2^ statistic (*I*^2^ 25% = low, 50% = medium, 75% = high; *p* < 0.10 indicating statistically significant heterogeneity). When heterogeneity was statistically significant, the source of heterogeneity was comprehensively identified through subgroup or sensitivity analyses. Publication bias was analyzed using a funnel plot and Egger's test when more than five eligible studies were included. Statistical significance was set at *p* < 0.05.

### Meta-Regression

To evaluate the potential source of heterogeneity in this meta-analysis, a meta-regression analysis was performed using a random-effects model. The selected variables were as follows: year of publication, number of patients, age, proportion of males, body mass index (BMI), proportion of comorbidities, baseline level of left atrial diameter (LAD), left ventricular ejection fraction (LVEF), and CHA2DS2-VASc score.

## Results

### Study Characteristics

**Figure 1:**
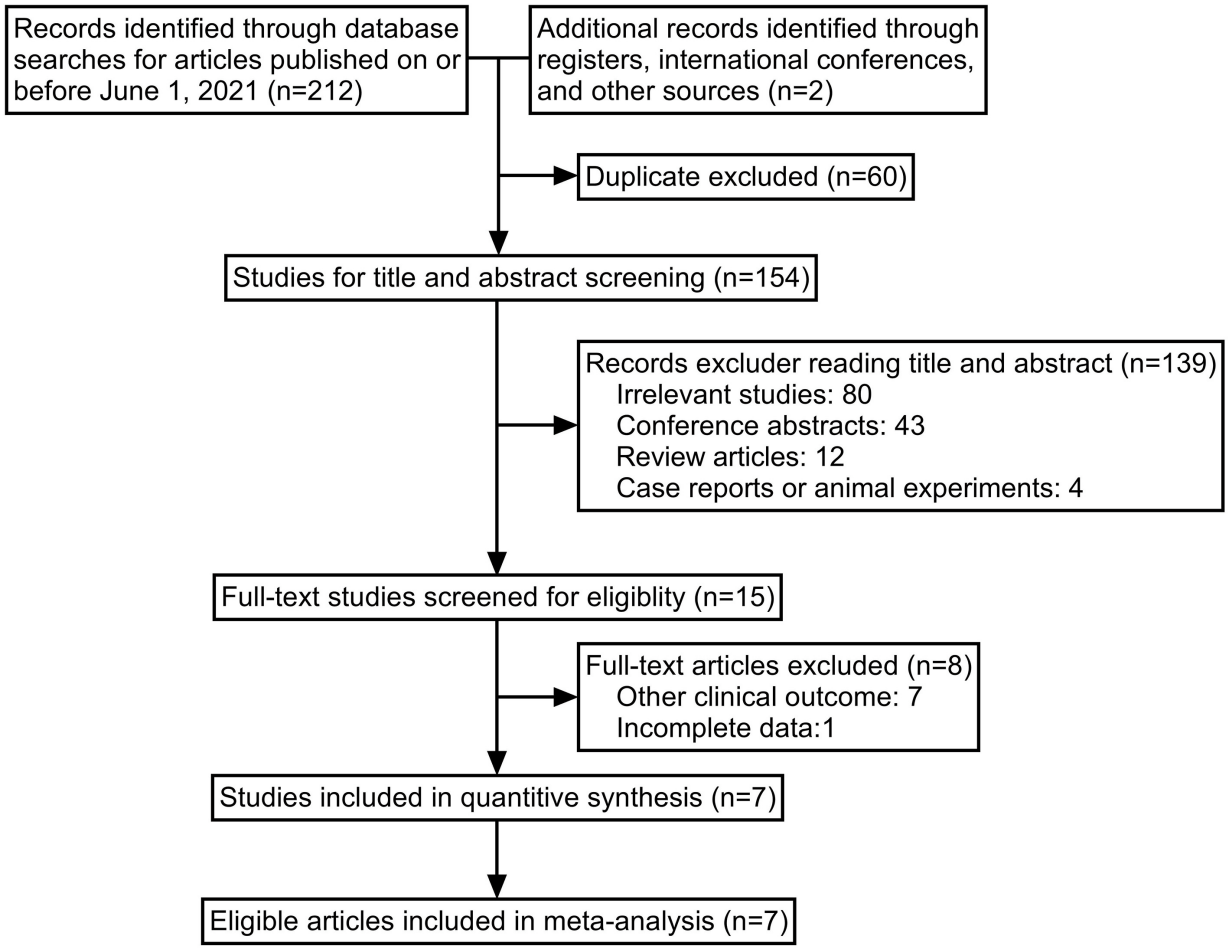
Flow diagram of the study selection process.

Following the literature search strategy, we retrieved 214 potentially relevant records. Sixty duplicate articles were eliminated, and 154 records were independently screened by title and abstract. A total of 139 articles were excluded, including obviously irrelevant articles (*n* = 80), conference abstracts (*n* = 43), review articles (*n* = 12), and case reports or animal experiments (*n* = 4). Furthermore, after carefully reviewing the remaining 15 results with full text, eight studies were excluded owing to incomplete data or irrelevant clinical outcomes. Therefore, seven studies that satisfied our inclusion and exclusion criteria were finally included in this meta-analysis (10–16). The stepwise selection process is illustrated in Figure 1.

Table 1 shows the main characteristics of the seven eligible studies, while Table 2 summarizes the baseline characteristics of the patients involved in the seven studies. We included both prospective and retrospective cohort studies involving 679 patients with AF who underwent HBA or CBA procedures.

**Table 1:** Main characteristics of 7 eligible studies included in the meta-analysis.

**No**.	**References**	**Type of study**	**Region**	**Number of Patients (HBA/CBA, *n*)**	**Follow up period**	**Evaluated parameters**
No. 1	Nagashima et al. (10)	Retrospective	Japan	37/37	11.8 m	Total mapping points, Touch-up ablation, Residual PV potential, Dormant conduction, AF Recurrence, Major complication, Antiarrhythmic therapy
No. 2	Nakamura et al. (11)	Prospective	Japan	58/65	46 ± 31 d	Procedure time, Touch-up ablation, Major complication
No. 3	Wakamatsu et al. (12)	Retrospective	Japan	46/46	12 m	Total mapping points, Touch-up ablation, Residual PV potential, Dormant conduction, AF Recurrence, Antiarrhythmic therapy
No. 4	Wakamatsu et al. (13)	Retrospective	Japan	79/79	18 m	Procedure time, Fluoroscopy time, Total mapping points, Touch-up ablation, AF Recurrence, Major complication, Antiarrhythmic therapy
No. 5	Hojo et al. (14)	Prospective	Japan	46/46	73 d	Procedure time, Fluoroscopy time, Touch-up ablation, AF Recurrence, Major complication
No. 6	Suruga et al. (15)	Retrospective	Japan	30/30	365 ± 102 d	Procedure time, Fluoroscopy time, Touch-up ablation, AF Recurrence
No. 7	Akita et al. (16)	Retrospective	Japan	40/40	12 m	Procedure time, Fluoroscopy time, Touch-up ablation, AF Recurrence

*AF, Atrial fibrillation; CBA, cryoballoon ablation; d, days; HBA, hot balloon ablation; m, month; PV, pulmonary vein*.

**Table 2:** Evaluated parameters, baseline characteristics of 7 eligible studies included in the meta-analysis.

**No**.	**References**	**Age (years)**	**Gender (male %)**	**BMI (kg/m^2^)**	**Paroxysmal AF (%)**	**HT (%)**	**DM (%)**	**Stroke or TIA (%)**	**HF (%)**	**Vascular disease (%)**	**LAD (mm)**	**LVEF (%)**	**CHA2DS2-VASc**
No. 1	Nagashima et al. (10)	62 ± 10.03	74.32	25 ± 4	62	59.46	24.32	13.51	9.46	4.05	40 ± 5.58	66 ± 11	2 ± 1.47
No. 2	Nakamura et al. (11)	65 ± 10	68.3	NG	95.1	46.3	17.1	6.5	3.3	4.9	38 ± 5	63 ± 8	1.9 ± 1.4
No. 3	Wakamatsu et al. (12)	62.63 ± 9.75	76.09	24.72 ± 4.26	63.04	48.91	19.57	9.78	6.52	5.43	39.22 ± 6.32	66.04 ± 8.76	1.78 ± 1.69
No. 4	Wakamatsu et al. (13)	64 ± 9.48	77.22	25 ± 4	0	53.16	16.46	12.03	13.92	5.7	41 ± 5.51	62 ± 12.00	2 ± 1.48
No. 5	Hojo et al. (14)	65.15 ± 9.31	81.52	23.95 ± 3.19	100	NG	NG	NG	NG	NG	NG	64.5 ± 6.36	1.75 ± 1.54
No. 6	Suruga et al. (15)	63.5 ± 10.43	85	24.55 ± 3.10	100	48.33	21.67	5	8.33	3.33	39.5 ± 5.16	65 ± 7.65	1.75 ± 1.54
No. 7	Akita et al. (16)	64.55 ± 9.47	78.75	24 ± 2.93	93.75	NG	NG	NG	NG	NG	38.5 ± 6.39	62 ± 5.93	NG

*BMI, Body mass index; DM, diabetes mellitus; HF, heart failure; HT, hypertension; LAD, left atrial diameter; LVEF, left ventricular ejection fraction; NG, not given; AF, paroxysmal atrial fibrillation; PV, pulmonary vein; TIA, transient ischemic attacks; VD, vascular disease*.

Most studies reported surgical parameters and clinical outcomes with short- and mid-term follow-up, including procedure time, fluoroscopy time, incidence of touch-up ablation (TUA), AF recurrence, and major complications. Several studies have also recorded total mapping points, residual PV potential, dormant conduction, and different antiarrhythmic therapies. All of the above results are included in this meta-analysis.

All seven studies were judged to be of high quality; the literature quality evaluation form is shown in Supplementary Table 2.

### Overall Meta-Analysis

#### Variables Related to TUA

**Figure 2:**
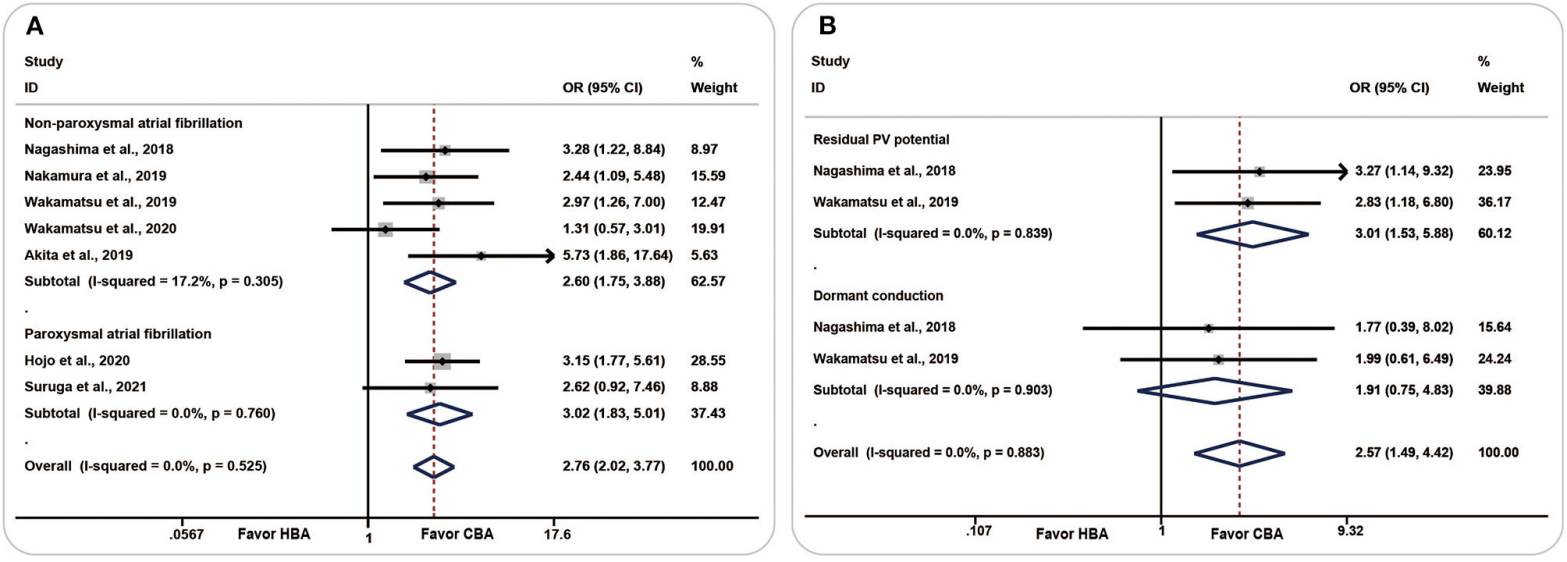
Forest plots showing the association between touch-up ablation and ablation strategy. **(A)** Touch-up ablation, **(B)** residual conduction. CBA, cryoballoon ablation; HBA, hot balloon ablation.

All seven included studies recorded the number of patients or PVs that required touch-up RF ablation, and the HBA group had a higher incidence of TUA [OR (95% CI) = 2.76 (2.02–3.77), *P* = 0.000] with low heterogeneity (*I*^2^ = 0.0%, *p* = 0.525). No significant change was observed in the subgroup analysis of paroxysmal AF and non-paroxysmal AF (Figure 2A).

Two studies evaluated the number of residual PV potentials and dormant conduction. The pooled analysis showed that, compared with the CBA group, more patients in the HBA group had residual PV potential [OR (95% CI) = 3.01 (1.53–5.88), *P* = 0.001, *I*^2^=0.0%, *p* = 0.839] and dormant conduction [OR (95% CI) = 1.91 (0.75–4.83), *P* = 0.174, *I*^2^ = 0.0%, *p* = 0.903] (Figure 2B).

Meta-regression regarding TUA of all involved studies revealed that the ablation strategy was not influenced by the publication year (*P* = 0.506), sample size (*P* = 0.129), age (*P* = 0.947), proportion of males (%) (*P* = 0.699), BMI (*P* = 0.242), proportion of comorbidity (%) (including paroxysmal AF, *P* = 0.154; hypertension, *P* = 0.981; DM, *P* = 0.319; stroke or TIA, *P* = 0.760; heart failure, *P* = 0.359; vascular disease, *P* = 0.433), baseline level of LAD (mm) (*P* = 0.252), LVEF (%) (*P* = 0.460), and CHA2DS2-VASc score (*P* = 0.317) (Supplementary Figure 1).

**Figure 3:**
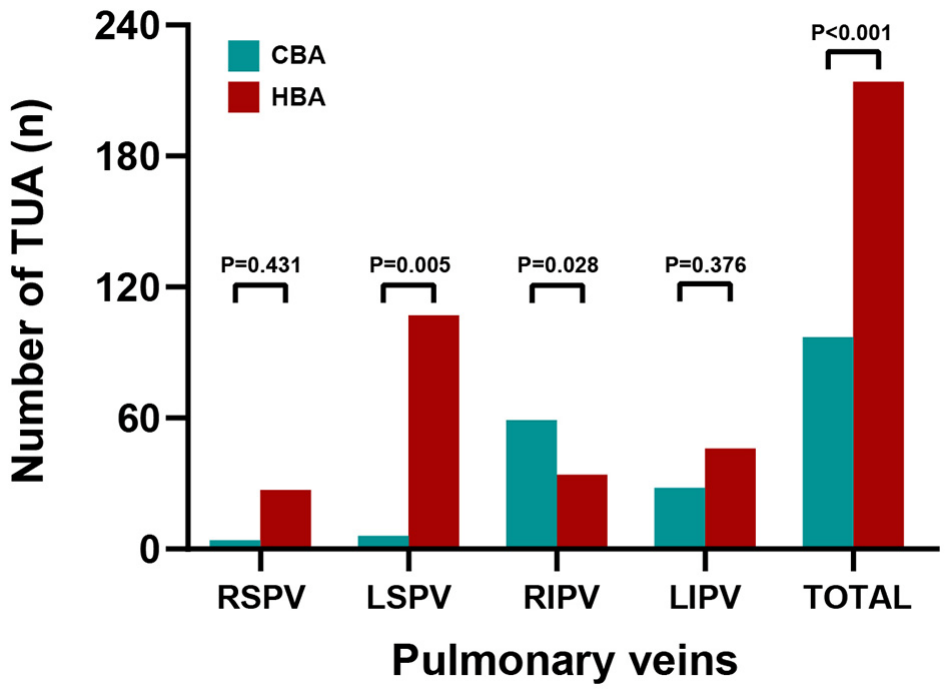
Diagram showing the touch-up ablation incidence of cryoballoon ablation and hot balloon ablation. A noticeable increase in the incidence of ablation was observed in the HBA group (*p* < 0.001). More touch-up ablations were applied in the RSPV, LSPV, and LIPV in patients with HBA. More ablations were performed in the RIPV in patients with CBA. Statistical significance was set at *p* < 0.05. CBA, cryoballoon ablation; HBA, hot balloon ablation; LIPV, left inferior pulmonary vein; LSPV, left superior pulmonary vein; RIPV, right inferior pulmonary vein; RSPV, right superior pulmonary vein.

The incidence of TUA following CBA and HBA procedures in PVs is shown in Figure 3. A noticeable increase in the incidence of ablation was observed in the HBA group (*p* < 0.001). More TUA was applied in the anterior carina and anterior ridge of the left superior pulmonary vein (LSPV) in patients with HBA. In contrast, more ablations were performed in the inferior aspect of the left inferior pulmonary vein (LIPV) and right inferior pulmonary vein (RIPV) in patients with CBA, especially for RIPV. A significant difference between the two procedures was observed in the LSPV (*P* = 0.005) and RIPV (*P* = 0.028).

**Figure 4:**
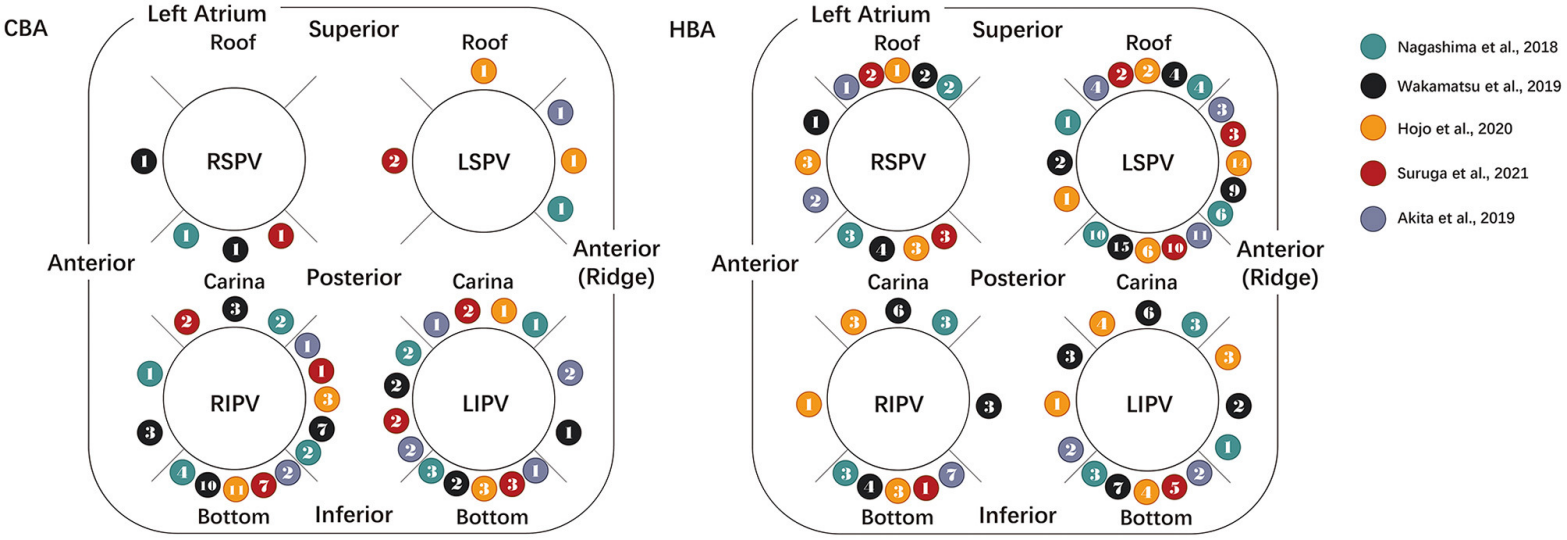
Diagram showing the distributions of the touch-up ablation sites. The distribution of touch-up ablation sites following CBA (left) and HBA (right) is summarized in the above diagram. The rightmost legends show the included literature represented by spheres of different colors. The number in the balls indicates the touch-up ablation incidences and sites reported in the literature. CBA, cryoballoon ablation; HBA, hot balloon ablation; LIPV, left inferior pulmonary vein; LSPV, left superior pulmonary vein; RIPV, right inferior pulmonary vein; RSPV, right superior pulmonary vein.

The distribution of TUA sites following HBA and CBA was reported in five studies and is summarized in Figure 4. In the HBA group, the left PVs had the highest incidence of TUA (LSPV, 50.0%; LIPV, 21.5%). The specific procedure was often required at the anterior aspect of the LSPV (carina, 48.6% of LSPV; ridge, 32.7% of LSPV) and the bottom of the LIPV (45.7% of LIPV). In the CBA group, ablation was often applied to the inferior PVs (RIPV, 60.8%; LIPV, 28.9%). Among the inferior PVs, the ablation incidences and sites were concentrated at the base (57.6% of the RIPV, 42.9% of the LIPV).

#### Procedure-Related Data

The procedure data for CBA and HBA were summarized in detail, including fluoroscopy time, total mapping points, and procedure time. A random-effects model was applied owing to significant heterogeneity.

The procedure time was reported in five studies, with the results showing that patients in the HBA group had a longer procedure duration than those in the CBA group [WMD (95% CI) = 9.69 (−2.78 to −22.16), *P* = 0.128]. No statistical difference was found between the two groups, and the heterogeneity was moderate (*I*^2^ = 62.6%, *p* = 0.030).

Four studies reported the results considering the fluoroscopic time. Compared with the HBA group, the pooled fluoroscopic duration was longer in the CBA group [WMD (95% CI) = −1.03 (−9.50 to −7.44)]; however, the results were statistically insignificant (*P* = 0.812) and the heterogeneity was considerable (*I*^2^ = 87.5%, *p* = 0.000).

The overall value of the total mapping points summarized from the three included studies was 525.79 [WMD (95% CI) = (−56.56 to −1108.13), *P* = 0.077, *I*^2^ = 90.5%, *p* = 0.000]. The above results indicated that the total number of mapping points was higher in the HBA group, with considerable heterogeneity.

### Lesion Size

Two studies reported the ablation lesion size; the pooled results showed that the lesion was larger in the CBA group [WMD (95% CI) = −2.22 (−16.52, 12.08), *P* = 0.761], although no statistical difference was observed. However, the heterogeneity was significant (*I*^2^ = 95.5%, *p* = 0.000). The above two studies also explored differences in lesion area (%). The pooled analysis revealed a neutral result [WMD (95% CI) = −0.16 (−19.96, 19.63), *P* = 0.987, *I*^2^ = 98.1%, *p* = 0.000]. The results are shown in Supplementary Figure 2.

### Sensitivity Analysis and Meta-Regression

To further assess the sources of intertrial heterogeneity, sensitivity analysis was performed by sequentially omitting each study.

**Figure 5:**
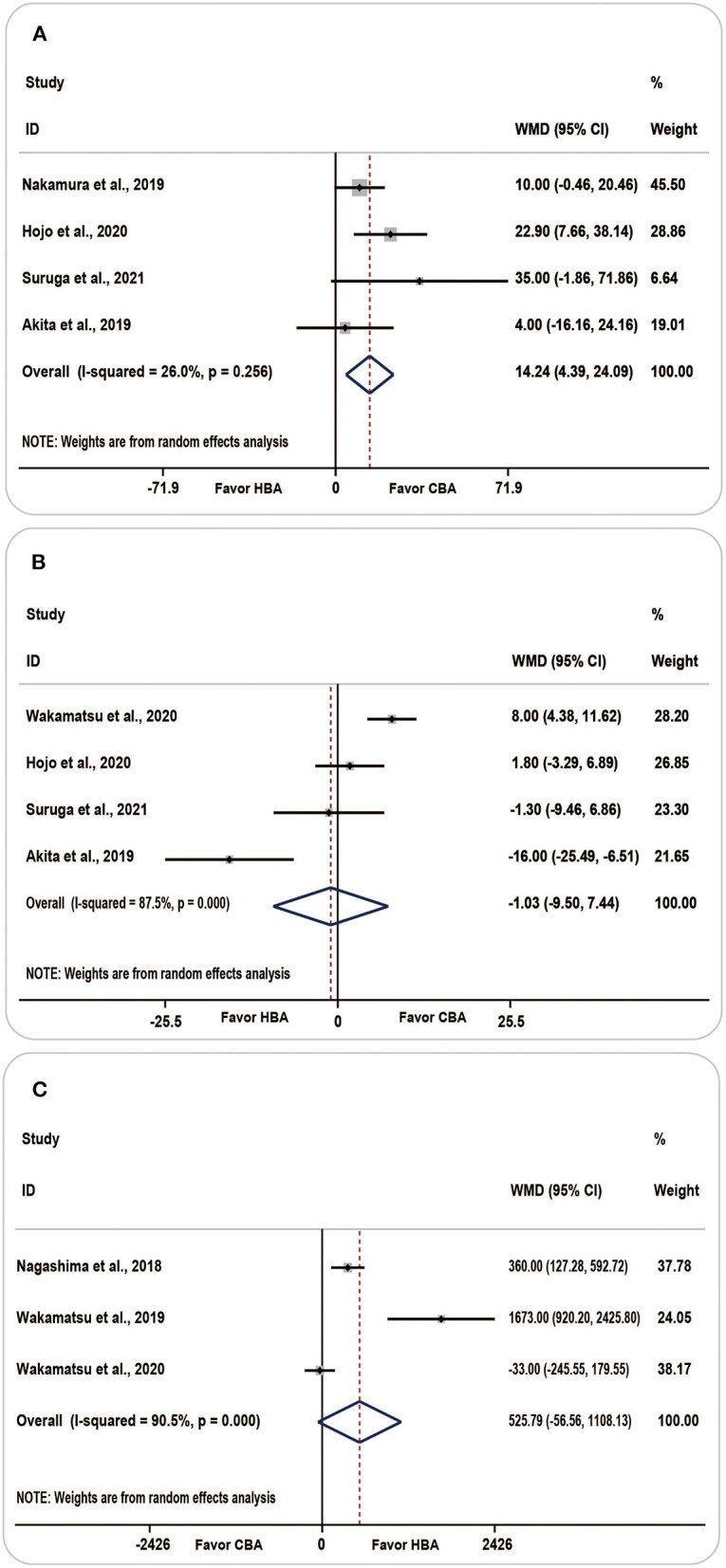
Forest plots showing the association between procedure-related parameters and ablation strategy. **(A)** Procedure time, **(B)** fluoroscopy time, **(C)** total mapping points. CBA, cryoballoon ablation; HBA, hot balloon ablation.

Considering the procedure time, based on the one-study removed model, we found that when we omitted the Wakamatsu's study, the analysis carried out on the four remaining studies revealed a noticeable decrease in heterogeneity from 62.6 to 26.0% [WMD (95% CI) = 14.24 (4.39–24.09), *P* = 0.005, *I*^2^ = 26.0%, *p* = 0.256] (Figure 5A). The results of the sensitivity analysis are shown in Supplementary Figure 3.

There was no significant change in heterogeneity following sensitivity analysis of fluoroscopic time and total mapping points, and the point estimate and 95% CI of the results were not appreciably altered. The forest plot of the fluoroscopic time and total mapping points is shown in Figures 5B,C.

Meta-regression was conducted to determine the potential source of heterogeneity. However, no potential source of heterogeneity was related to fluoroscopy time, total mapping points, and procedure time.

### Fluid Injected Into the HBA

**Figure 6:**
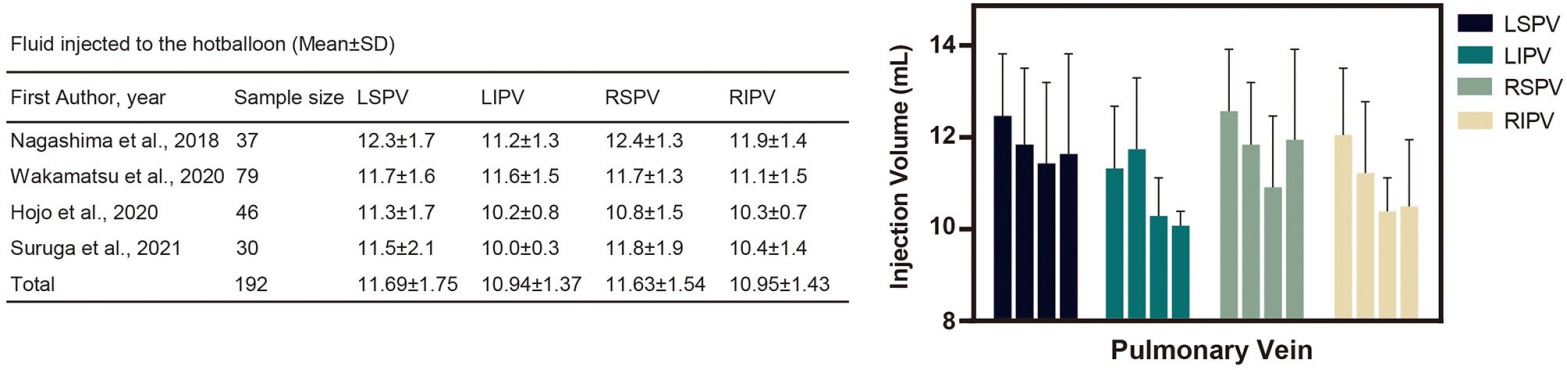
Fluid injected into the hot balloon (Mean ± standard deviation). The above table and diagram summarize the amount of fluid injected into the left atrial pulmonary veins during the hot balloon procedure. As shown in the chart, the LSPV and RSPV received more fluid injections during the procedure. LIPV, left inferior pulmonary vein; RIPV, right inferior pulmonary vein; RSPV, right superior pulmonary vein; RIPV, right inferior pulmonary vein; SD, standard deviation.

Four studies reported the fluid injection volume of HBA for PV occlusion. The amount of injected fluid is summarized in Figure 6; among the 4 PVs, the left superior and right superior PVs are more likely to be agitated with a higher volume of fluid.

### Clinical Outcomes

**Figure 7:**
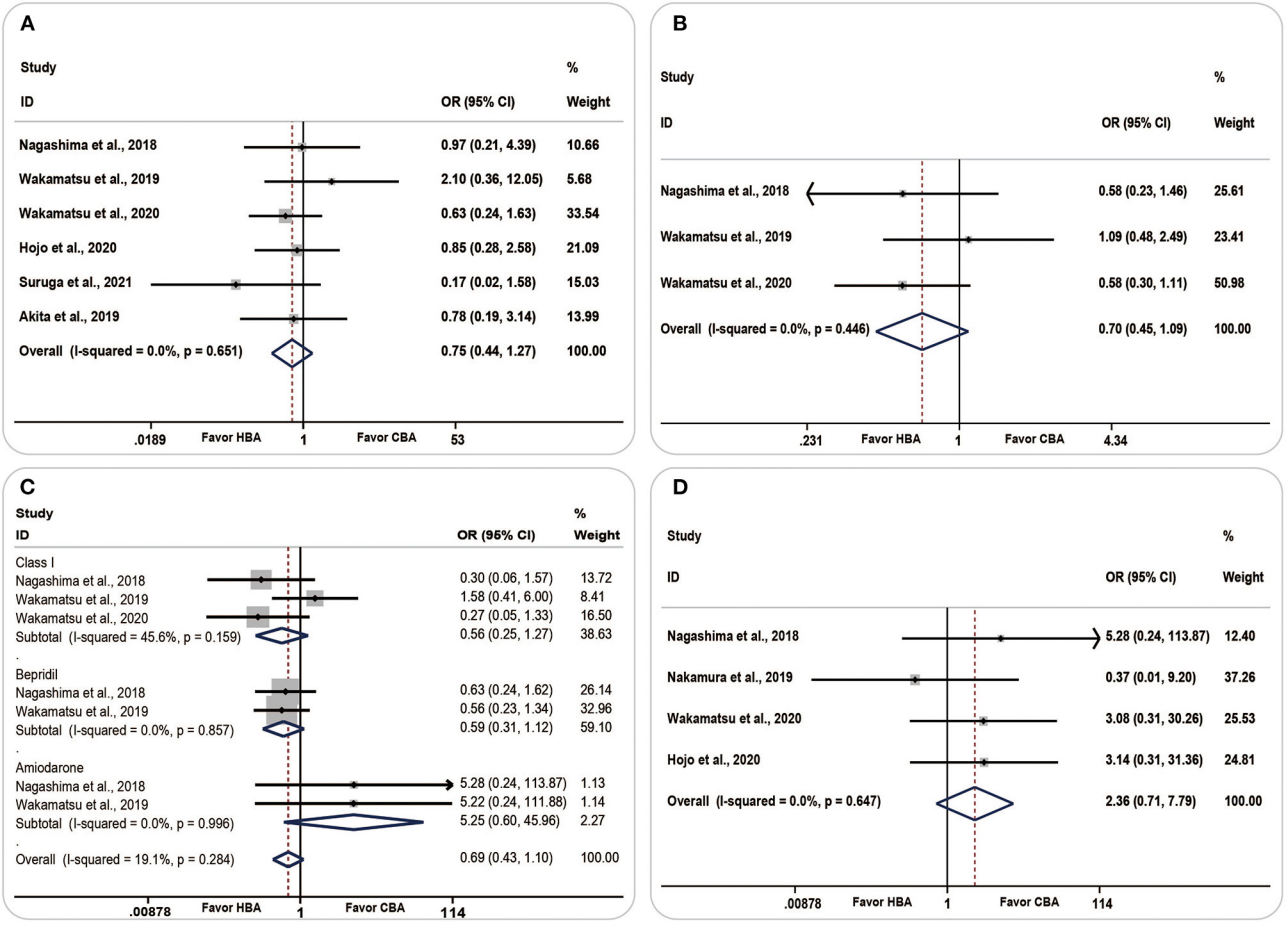
Forest plots showing the association between clinical outcomes and ablation strategy. **(A)** Atrial fibrillation recurrence, **(B)** antiarrhythmic therapy, **(C)** antiarrhythmic drug therapies in detail, **(D)** complications. CBA, cryoballoon ablation; HBA, hot balloon ablation.

Six studies analyzed the AF recurrence and found that more patients in the CBA group were likely to have AF occurrence [OR (95% CI) = 0.75 (0.44–1.27), *P* = 0.281, *I*^2^ = 0.0%, *p* = 0.651] and accepted more antiarrhythmic drug therapy [OR (95% CI) = 0.70 (0.45, 1.09), *P* = 0.114, *I*^2^ = 0.0%, *p* = 0.446], although the result was statistically insignificant (Figures 7A,B).

There was no significant change in the AF recurrence during the subgroup analysis of paroxysmal or non-paroxysmal AF and follow-up periods (Supplementary Figures 4A,B).

Among all the antiarrhythmic drug therapies, both class I drugs and bepridil were more likely to be used in the CBA group [OR (95% CI) = 0.56 (0.25–1.27), *P* = 0.165, *I*^2^ = 45.6%, *p* = 0.159; OR (95% CI) = 0.59 (0.31–1.12), *P* = 0.107, *I*^2^ = 0.0%, *p* = 0.857, respectively]. Amiodarone was more frequently used in the HBA group [OR (95% CI) = 5.25 (0.60–45.96), *P* = 0.134, *I*^2^= 0.0%, *p* = 0.996] (Figure 7C).

Complications were compared in four studies. The pooled analyses showed a higher prevalence of complications in the HBA group [OR (95% CI) =2.36 (0.71–7.79), *P* = 0.160, *I*^2^ = 0.0%, *p* = 0.647]; however, the result was insignificant (Figure 7D).

### Publication Bias

We performed a funnel plot and Egger's test to examine the publication bias. Egger's test resulted in a *P*-value of 0.773, suggesting no significant publication bias in this meta-analysis (Supplementary Figure 5).

## Discussion

### Main Findings

This meta-analysis included seven studies with a total of 679 patients. To the best of our knowledge, no previous meta-analysis has compared the characteristics of the clinical outcomes between these balloon modalities.

The main findings were as follows:

1) Compared with CBA, patients in the HBA group had more residual conduction, higher incidence of TUA and longer procedural time,
2) The most frequent sites of TUA were the left superior PVs in the HBA group vs. the right inferior PVs in the CBA group, and
3) The clinical outcomes during the mid-term follow-up between the HBA and CBA groups were comparable. Although patients in the CBA group had a higher risk of AF occurrence and accepted more antiarrhythmic therapies, the HBA group had more surgical complications; the between-group difference was insignificant.

### Residual Conduction and TUA

As revealed in this study, more cases with residual PV potentials and dormant conduction were observed in the HBA group, which required a significantly higher incidence of additional touch-up RF ablation. Based on the findings of the present studies (10, 17, 18), the unequal comparison of the TUA between the HBA and the CBA could be attributed to characteristic balloon compliance and ablation size.

Considering the compliance of the balloon modalities, Yamasaki et al. reported that the hot balloon (HB) had better compliance and adjustability during the procedure, in which the size and shape of the balloon were easily altered to accommodate variations in the PV geometry and achieve optimal occlusion and isolation of the PVs (18). By fluently inflating inside the PV ostium, the HB could facilitate the occlusion of the more distal and deeper portions of the PVs (10). However, this elastic feature could lead to a suboptimum contact area of the PV ridge, which corresponds to the balloon ablation lesion, and results in inadequate occlusion of the PV antrum. These could be indicative of the need for additional RF ablation of the ridge following the HB system.

Compared to the HB, the cryoballoon (CB) was non-conforming and did not vary in size and shape (17). This feature stiffened the CB during energy deliveries and allowed it to expand outside the PV before advancing to the PV port, resulting in distension of the PV ridge and a larger ablation area of the antrum.

Additionally, the left atrium (LA) ablation lesion size could also be related to the need for TUA. It has been proven that balloon-based pulmonary vein isolation (PVI) could produce a wider ablation lesion than standard RF-based ablation (19). However, studies have shown that CBA might produce an even larger LA lesion size, lesion width, and lesion gap than the HBA (10, 16), which is consistent with the results of our analysis. This could be explained by the relatively large size of the second-generation CB, which had a large contact area and was capable of creating a larger freezing surface. In contrast to the cryothermal energy, the further the HB expanded compared to the standard inflated size; the less thermal energy it could deliver to the distal tissue, which could explain the narrow lesion created by the HBA. Although the lesion of the HB system was narrower, its durability rate was as constant as that of CBA. However, owing to limited research focusing on lesion differences between the two procedures, more studies are warranted for an incontestable conclusion.

Wakamatsu et al. (12) stated that HBA often could not provide ideal occlusion because the HB surface did not adhere well to all the tissues of the PV walls during PVI. This problem could not be solved by further advancing the enlarged HB to distend the orifice or extend the contact area since the overinflation could lead to dislodgment of the catheter from the PV ostium.

In contrast to HB, the stiffer CB remained fixed around the antrum, allowing for better and larger balloon surface–tissue contact by covering both small and large PVs, allowing for extensive PV distention, and creating wide-area antral lesion sets efficiently. This conclusion was consistent with a previous study showing that the area coverage in the left atrial was more stable, and the lesion creation of the CB was relatively larger (4). Therefore, the smaller isolation areas created by the HB systems could lead to further TUA.

The learning curve of HBA could also influence the incidence of TUA. The HB system has recently been introduced into the AF treatment and has not been widely applied in the arrhythmia centers, which limits the further development of the procedure. As Nagashima et al. (4) suggested, an improved ablation outcome and less demand for TUA were observed following an accumulation of HB experience. However, the results have been reported for experienced CBA operators. This phenomenon suggests the influence of a learning curve on the requirement of TUA in HBA surgery.

### Distribution of the TUA Sites

Apparently, in the above diagram, in the CBA group, the superior PVs and the anterior aspects of the inferior PVs were less needed for additional touch-up RF ablation. The procedure was prominently distributed in the inferior aspect of the RIPV and LIPV, especially for the RIPV. The potential reason for the above consequences could be the imperfect alignment of the inferior PVs (10, 12). The angle of the inferior PVs, the short distance between the puncture site and the vein, or the tight space between the dorsal vertebrae and the vein could lead to unstable and inadequate contact between the CB and the LA myocardium. During the inferior PVs' balloon inflation, the CB stretched in the superior direction and led to suboptimal contact and less PV distention in the inferior aspect, resulting in limited frozen tissue and smaller PV ostial lesions. Lesion creation in the superior and carina regions is particularly effective. Compared with superior PVs, the inferior PVs were relatively slimsy and small, resulting in a greater contact area between the balloon surface and blood flow. Owing to the CB, the freezing temperature was markedly influenced by the surrounding blood flow, and the larger surrounding area counteracted the optimal tissue freezing. These balloon features could cause specific characteristics and distributions of CB lesions.

Interestingly, among the HBA procedures, the right PVs were less likely to receive TUA, and the applications were frequently required in the anterior carina and anterior ridge of the LSPV, which was obviously different from the CBA procedure. This could be related to two reasons. A possible explanation for the group difference in TUA at the anterior ridge could be related to the compliance mentioned above. A dominant TUA site was observed near the anterior ridge in the HBA group; compared with HB, the characteristically non-compliant CB could distend the ridge better and produce a more extensive isolation area. In contrast, the adjustable HB inflated into the ostium easily so that the distension of the PV antrum was inadequate, resulting in more TUA near the anterior ridge. Another possible explanation for the distribution differences was the anatomic features. In the HBA procedure, the frequency of TUA was especially higher in the anterior ridge and carina areas of the LSPV. This result could perhaps be explained by the anatomic features, such as the left atrial wall thickness. The mechanism of lesion creation underlying the HBA was capacitive energy transfer, which was notably affected by the thickness of the LA myocardial tissue. Under temperature control, the heating fluid was agitated to ablate the LA tissue by conductive heating energy from the balloon surface and finally create a wide planar antral isolation area. The temperature decreases gradually as the endocardial tissue moves further away from the balloon surface. Among the PVI procedures, the anterior ridge and carina area of the LSPV is the thickest area, which profoundly cuts down the energy penetration depth, hinders the creation of a transmural lesion, and concentrates more PV reconnections (20). Hojo et al. reported that a balloon temperature of 70–73°C could reduce TUAs, thereby improving the outcomes (14).

Considering the above discussion focusing on the characteristics of TUA, several aspects are worth learning to guide future clinical applications. In terms of HBA, as an anatomy-dependent procedure, broader coverage between the balloon and PV antral tissue was crucial for optimal ablation. Further investigation and development concerning the techniques to enhance heat transfer and improve balloon-tissue contact are warranted. Second, more attention should be paid to the anterior ridge and the LSPV area, which warrants better balloon contact and deeper capacitive-type heating to achieve wider and more durable lesions. As for the CBA, in order to perform preferable ablation, precise atrial septal puncture site, the inflation angle, and the dorsal vertebrae space are warranted. Further, longer freezing times could be required in the inferior aspect of the PVs to realize optimal ablation lesions, decrease the TUA rate, shorten the procedure duration, and increase the PVI success rate.

### Ablation Outcomes

Our study compared the clinical outcomes reported in the published literature. First, the between-group difference in complications did not significantly differ between the modalities. Although several published articles reported procedural complications, such as fatal atriobronchial fistula formation and ice formation, which were closely related to the balloon location (21, 22), were higher in the CBA and the PV stenosis was higher in the HBA, our pooled analysis revealed that the difference was comparable. To avoid HB complications, a more appropriate balloon position and added balloon injection volume should be considered (21). As the clinical implication of CBA, a more proximal location between CB and LSPV and relatively counterclockwise rotation are strongly recommended to optimize the cryothermal injury.

Furthermore, middle-term outcomes, including AF occurrence and antiarrhythmic drug therapy, also revealed a similar incidence. Further, subgroup analysis revealed that AF occurrence was not significantly associated with persistent AF percentage or follow-up length.

Interestingly, even though HBA had more residual conduction and a higher TUA rate, the AF occurrence was comparable to that of the CBA procedure. A possible explanation could be that the soft HB can be modulated to fit the antral region, ablated inside the PV orifice, and created more lesions in the PVs, which covered the shortage of inadequate antrum ablation. Although the HBA lesions were small, they were as durable as those achieved by CBA (23). Another underlying reason could be related to the study design. Since the balloon-based technique is relatively novel, there is a lack of larger prospective multicenter randomized studies with long-term follow-up. Future comparative studies are warranted to reveal the differences, elucidate the clinical efficacy, and validate these two approaches.

## Limitation

Certain limitations of this study need to be acknowledged. First, potential confounding factors were not easy to control in a non-randomized comparative study. Although most studies have matched the propensity score between the two groups, unknown confounding factors could still exist. In some cases, meaningful information, such as the CHA2DS2-VASc score and echocardiographic parameters, could be missing. Second, this meta-analysis included only Japanese studies. The results could differ according to the race and country; the relatively small sample size could limit the representativeness of the samples, which could bias our conclusions. Finally, the follow-up period of some included studies was relatively short, and some patients might not have reached clinical endpoints, which could have influenced the clinical outcomes. However, as the first meta-analysis to investigate the difference in procedure-related data and clinical outcomes between the two balloon systems, we believe that the results of our study could provide guidance for clinical applications and promote further technological developments.

## Conclusion

To summarize, this meta-analysis shows that both HBA and CBA are practical ablation approaches for the treatment of AF. Patients who received HBA had a higher incidence of TUA and longer procedure time. The clinical outcomes during the mid-term follow-up were equivalent between HBA and CBA.

## Data Availability Statement

The original contributions presented in the study are included in the article/Supplementary Material, further inquiries can be directed to the corresponding author/s.

## Author Contributions

XP contributed to the literature search, data extraction, data analysis, and manuscript drafting. XLiu and HT contributed to the data extraction and manuscript drafting. YC contributed to the data extraction and data analysis. XLi contributed to the study conception, provided statistical expertise, revised the paper, and approved the final manuscript. All authors participated in the interpretation of the results, critical revision of the manuscript, contributed to the article, and approved the submitted version.

## Conflict of Interest

The authors declare that the research was conducted in the absence of any commercial or financial relationships that could be construed as a potential conflict of interest.

## Publisher's Note

All claims expressed in this article are solely those of the authors and do not necessarily represent those of their affiliated organizations, or those of the publisher, the editors and the reviewers. Any product that may be evaluated in this article, or claim that may be made by its manufacturer, is not guaranteed or endorsed by the publisher.

## Abbreviations

AF: atrial fibrillation
BMI: body mass index
CB: cryoballoon
CBA: cryoballoon ablation
CI: confidence interval
DM: diabetes mellitus
HB: hot balloon
HBA: hot balloon ablation
LA: left atrium
LAD: left atrial diameter
LIPV: left inferior pulmonary vein
LSPV: left superior pulmonary vein
LVEF: left ventricular ejection fraction
NOS: Newcastle-Ottawa Scale
OR: Odds ratio
PV: pulmonary vein
PVI: pulmonary vein isolation
RF: radiofrequency
RIPV: right inferior pulmonary vein
RSPV: right superior pulmonary vein
SD: standard deviation
TIA: transient ischemic
TUA: touch-up ablation
WMD: weighted mean difference.
