# Supplementary material for: Effects of Hot Balloon vs. Cryoballoon Ablation for Atrial Fibrillation: A Systematic Review, Meta-Analysis, and Meta-Regression
Source: Front Cardiovasc Med. 2021 Dec 15;8:787270. doi: 10.3389/fcvm.2021.787270 (PMC8714841; doi:10.3389/fcvm.2021.787270)

**Supplementary Materials**

**Supplementary Table. 1 Search Strategies**

The search included: PubMed, Embase, Cochrane Library, Web of Science, ClinicalTrial.gov, and medRxiv: The search date was from the inception through June 2021.

**Supplementary Table. 2** Assessment of study quality using the Newcastle-Ottawa scale.

**Supplementary Figure. 1** A meta-regression regarding touch-up ablation and sample size (P=0.129).


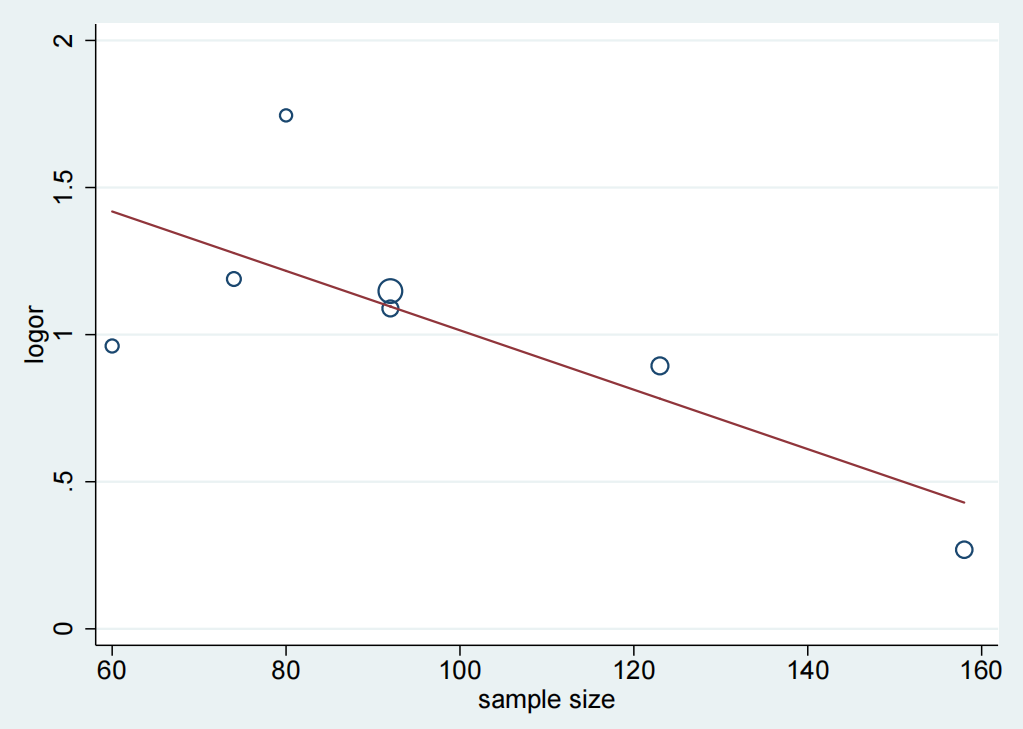


**Supplementary Figure. 2** Meta analysis of (A) ablation lesion size (cm^2^) and (B) lesion area (%).

*CBA, cryoballoon ablation; HBA, hot balloon ablation.*

**Supplementary Figure. 3** Sensitivity analysis of procedure time. Wakamatsu's study has been shown to affect the heterogeneity results significantly.


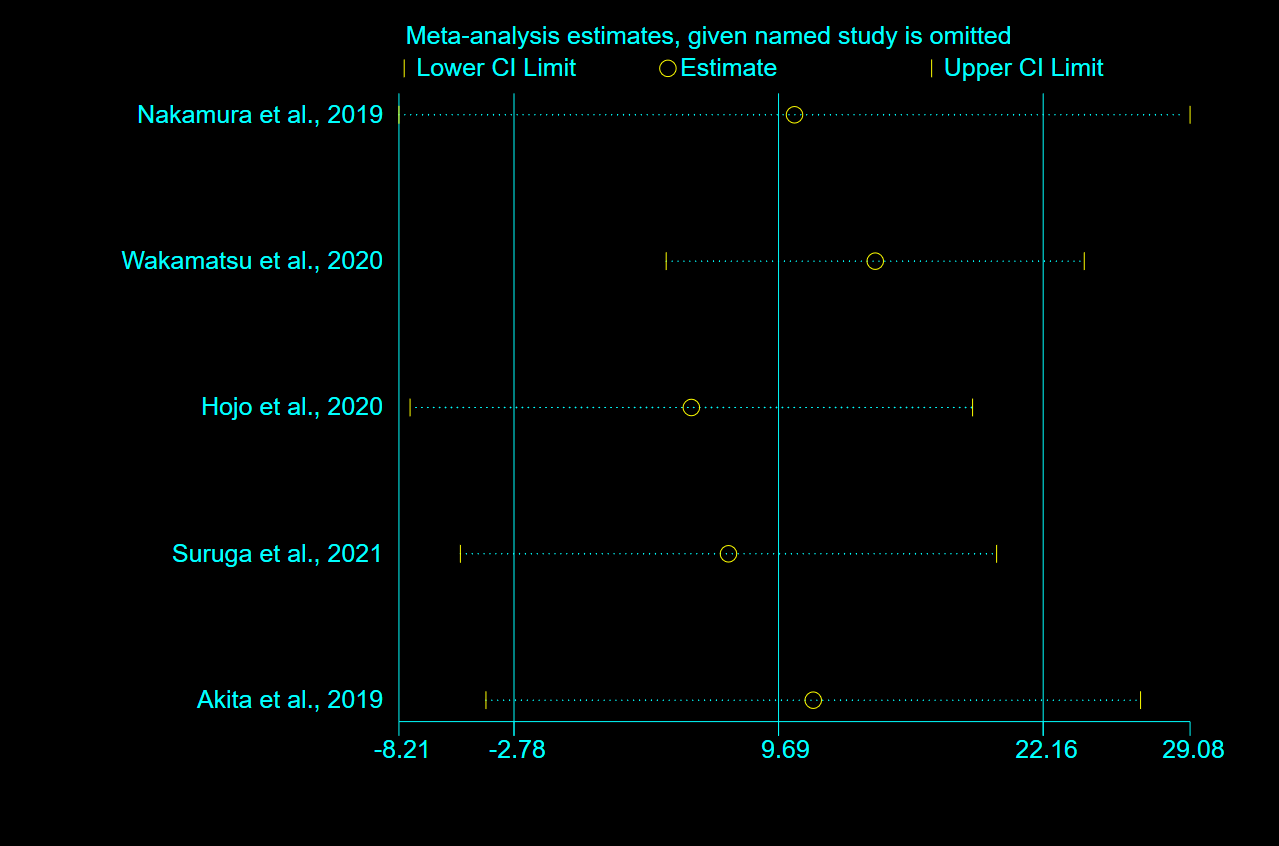


**Supplementary Figure. 4** Subgroup analysis of AF recurrence regarding (A) paroxysmal or non-paroxysmal AF and (B) follow-up periods.


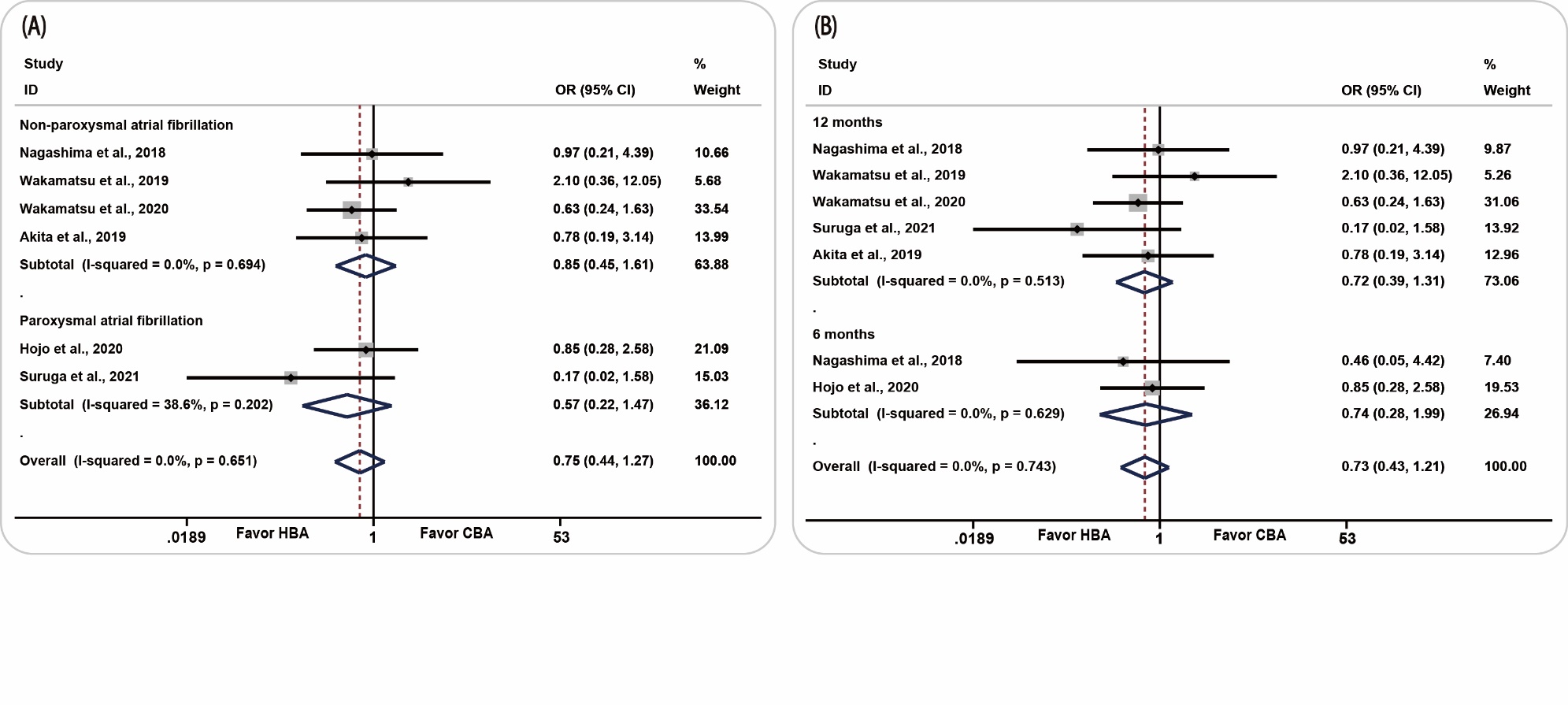


*CBA, cryoballoon ablation; HBA, hot balloon ablation.*

**Supplementary Figure. 5** Publication Bias-Funnel plots for studies evaluating touch-up ablation.


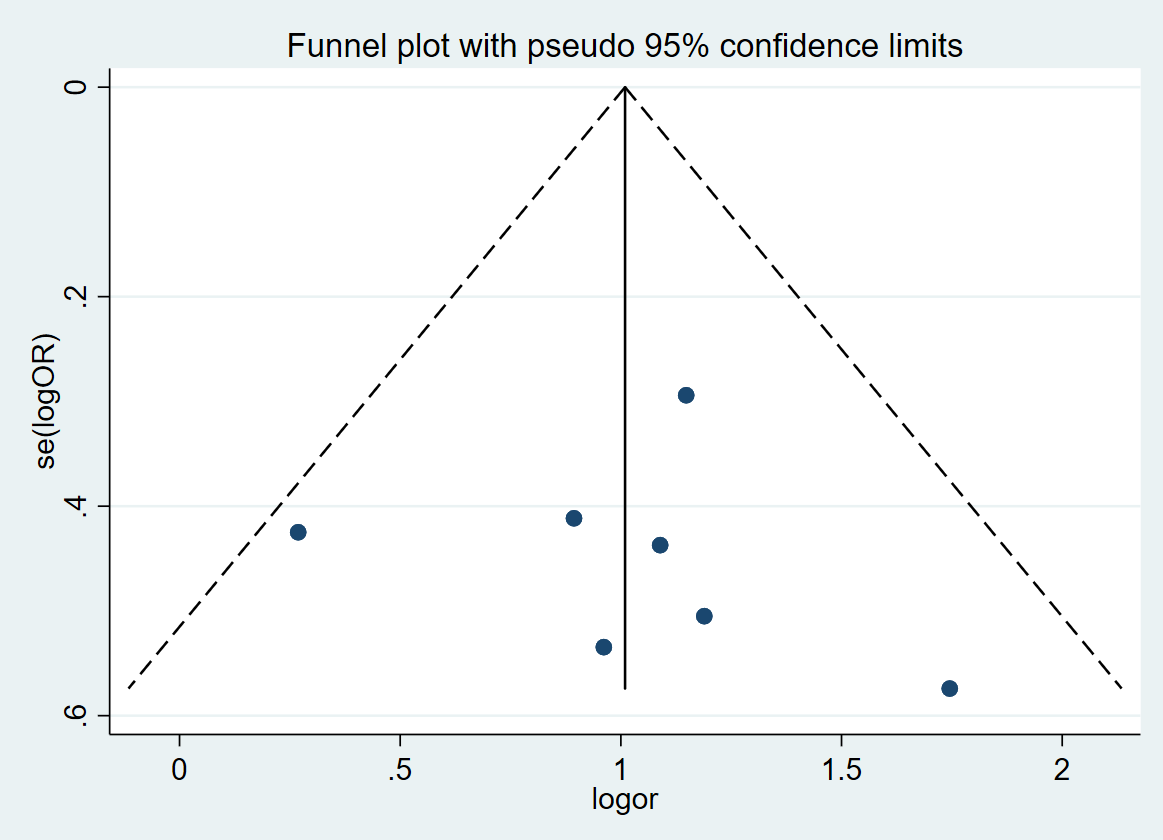

Supplement: Supplementary file 1 [file Data_Sheet_1.docx]
